# Information Complexity and Behavior Intention to Prescribe Antibiotics Based on the Antimicrobial Susceptibility Testing Report: The Mediating Roles of Information Overload and Attitude

**DOI:** 10.3389/fphar.2021.778664

**Published:** 2021-11-10

**Authors:** Ying Wang, Xinping Zhang, Qian Zhou, Xiaojun Xu, Xiaofeng Liu, Shaohui Lu

**Affiliations:** ^1^ School of Medicine and Health Management, Tongji Medical College, Huazhong University of Science and Technology, Wuhan, China; ^2^ The First Affiliated Hospital of Gannan Medical University, Ganzhou, China

**Keywords:** antimicrobial susceptibility testing report, antibiotic prescription, information complexity, behavior intention, information overload, attitude

## Abstract

**Background:** The antimicrobial susceptibility testing (AST) report has guiding significance for physicians to prescribe antibiotics. This study aims to examine the effect of the AST report information complexity on physician’s intention to prescribe antibiotics based on the AST report, as well as the mediating role of information overload and attitude.

**Methods:** A cross-sectional study conducted on 411 physicians in a general hospital in China in July 2021. Data were collected by a self-reported questionnaire. A serial multiple mediation model was tested to explore the sequential causality between the information complexity of the AST report, information overload, attitude, and behavior intention to prescribe antibiotics based on the AST report by using the SPSS macro PROCESS program.

**Results:** Information complexity, information overload, attitude and behavior intention were significantly correlated (*p* < 0.01). Information complexity can not only have a direct positive impact on the intention to prescribe antibiotics based on the AST report (effect = 0.173; SE = 0.044; Boot95%CI: LL = 0.089, UL = 0.260), but also have an indirect impact on behavior intention through the independent mediating role of information overload (effect = 0.025; SE = 0.011; Boot 95%CI: LL = 0.008, UL = 0.050) and the independent mediating role of attitude (effect = 0.130; SE = 0.025; Boot 95%CI: LL = 0.086, UL = 0.180), while the chain of information overload and attitude played a masking effect between information complexity and behavior intention (effect = −0.013; SE = 0.004; Boot 95%CI: LL = −0.023, UL = −0.005).

**Conclusion:** The increase in information complexity can encourage physicians to prescribe antibiotics based on the AST report, information overload and attitude can promote this effect. It is necessary to provide physicians with sufficient information to prescribe antibiotics without increasing the burden on them. At the same time, publicity and standardized training should be conducted for physicians to interpret the AST report better and faster.

## Introduction

The current situation of Antimicrobial resistance (AMR) is severe and the main reason is irrational use of antibiotics. Rational prescription of antibiotics by physicians is the key to controlling the hazards of AMR and is of importance to public health (Hashemi, et al., 2013). Antibiotic prescription behavior is a complex process and is affected by many factors such as physicians’ attitude and time pressure, patients’ diseases and expectations, and institutes and governments’ antibiotic-relevant policies and guidelines and their implementation (Teixeira Rodrigues et al., 2013).

To curb AMR, the World Health Organization and the European Commission require strengthening laboratory capacity building to promote the rational use of antibiotics (Castro-Sánchez et al., 2018; WHO, 2019). A Study showed that laboratory capability on AMR characterization plays a significant role in reducing the rate of resistance (Cui et al., 2019). Antimicrobial susceptibility testing (AST) is one of the most important functions of a clinical microbiology laboratory, and the AST report plays a vital role as the result of AST (Turnidge, 2015), so it is an important manifestation of clinical microbiology laboratory capacity. As an important basis for clinical decision-making, the AST report has guiding significance for physicians to prescribe antibiotics (Drobniewski et al., 2015). Evidence showed that the method of microbiological laboratory reporting test results, as well as selective reports and instructions on how to interpret the results, may have an important impact on physicians’ prescription habits (Morency-Potvin et al., 2016; Daley et al., 2018). Therefore, it is worthy to study the behavior intention of physicians to prescribe antibiotics based on the AST reports and its influencing factors.

Granato (1993) found that although most physicians believe that AST reports are the most important information generated by microbiology laboratories, but they often ignore the AST results and prefer empirical therapy when determining to prescribe antibiotics and one reason for that situation is information overload. Information overload refers to the peoples’ information processing needs are greater than information processing capabilities in the process of information search as the individual’s information processing capacity is limited, it will cause decision-making to become chaotic and inaccurate (Jacoby et al., 1978). A study on reports of sensitive *Escherichia coli* for blood and urine cultures showed that 65 and 63% of reports have information overload (Neu, 1978). This may cause physicians to fail to understand the report correctly or have a negative attitude towards the report, thus failing to use the report reasonably, and ultimately leading to incorrect diagnosis and irrational antibiotic prescription (Steffee et al., 1997; Graham et al., 2019).

The information complexity of the AST report may be an important reason for the information overload. Kroth et al. (2019) showed that the report usually marks the detected abnormality and the normal result at the same time, occupying the same space and degree of prominence, providing too much worthless information, resulting in information overload. Previous studies have proved that information complexity is a central proximal cause of information overload (Antoni and Ellwart, 2017; Graf and Antoni, 2021), which in turn lead to further experiential and behavioral consequences (Bock et al., 2010). However, the influences of information complexity on physicians’ intention to approach the AST report are not well understood. The information complexity of the AST report might have both positive and negative effects on the intention to approach the AST report (Huang, 2000).

There have been many studies on the impact of information complexity on information overload, as well as the impact of information overload on information users’ attitudes, behaviors, and behavior intentions in various fields. However, there are few studies on the relationship between information complexity and behavior intentions, especially on the influence mechanism of antibiotic prescription behavior intention from the perspective of information complexity.

This study aims to examine the association between the information complexity of AST reports and the physician’s intention to prescribe antibiotics based on the AST report, as well as the underlying mechanism–the mediating roles of information overload and attitude.

### Information Complexity and Behavior Intention to Prescribe Antibiotics Based on the AST Report

Complexity refers to the number of information features (Campbell, 1988), the complexity and similarity between choices increase the number of available options, fully loading a person’s working memory and cognitive capacity (Messner and Wanke, 2011). Paul and Nazareth (2010) defined information complexity as the amount of information and the diversity of information. The problem information complexity in health care has been mentioned by many of the health orientated papers (Hall and Walton, 2004), but there is a lack of research on the information complexity in the AST report and its impact on the behavior intention of antibiotic prescription. We refer to researches on the relationship between information complexity and behavioral intentions in other fields such as online shopping and social media use.

When the information exposed to users is too complex, people may become overwhelmed and their behavior intention will be affected. Consumers’ ability to search for information in the process of choosing products is limited. When they perceive complexity, consumers may feel confused and do not know how to choose (Huffman and Kahn, 1998). Kwon et al. (2015) and Li (2016) believe that all aspects of online review information on online shopping sites reflect the complexity and complexity is one of the most important factors leading to consumers’ perception of information overload. Once perceived information overload, consumers will feel overwhelmed and their intention to purchase online will decrease.

However, some studies have shown that information complexity has a positive effect on behavior intention. In the online shopping environment, Donovan and Rossiter (1982) found a beneficial effect of information complexity and novelty on the desire to stay longer in conventional retail environments, Huang believes that this kind of information complexity will make respondents expect greater variety of goods available, thereby promoting purchase intention, although this may be an impulse purchase (Huang, 2000).

Physicians are often faced with a large amount of patient information in paper or electronic medical records (Fuat et al., 2003), Providing more information may improve the quality and efficiency of clinical decision-making and improve the level of patient care (Laker et al., 2018). But there is also evidence that providing more information may not achieve these goals, and worse, it may contribute to errors in clinical decision-making (Ash et al., 2004). An important reason is that when the complexity of the information provided reaches a certain level, clinicians will be at a loss when they cannot process all the information in the system (Singh et al., 2013).

The AST report is a product that integrates various information from clinical and microbiological laboratories, physicians need to process this complex information, and make the best choice among the many drugs reported in combination with the patient’s situation. When the complexity of the information in the AST report increases, clinicians may believe that the AST report provides a more professional and scientific basis for prescription and then trust the AST report, as well as be willing to prescribe the antibiotics based on the report. However, some researchers also pointed out that the microbiology report is not like most biochemical or hematology reports which has absolute validity (Ackerman et al., 1980), there are many uncertainties, and professional microbiology knowledge is required for physicians to correctly interpret the test results. Once the laboratory report contains too detailed laboratory information, it will cause confusion and misinterpretation to physicians and lead to improper responses (Cunney and Smyth, 2000; Morency-Potvin et al., 2016), for example, the attitude toward the report may become negative, or show distrust, thereby reducing the intention to use AST report information as a reference for prescribing antibiotics.

Accordingly, it is hypothesized that the information complexity of AST reports is positively associated with the physician’s intention to prescribe antibiotics based on the AST report.

### The Roles of Information Overload and Attitude

Information overload is described as a stress condition caused by information characteristics (Graf and Antoni, 2021). Information complexity, as one of the important information characteristics (Li, 2016; Graf and Antoni, 2021), is an important influencing factor leading to information overload. When the amount of information is large, as well as the information features and dimensions, the complexity of the information will increase, especially when information is unfamiliar, information overload is more likely to happen, which will affect the individual’s psychological state and behavioral intentions (Otondo et al., 2008).

Paul and Nazareth (2010) pointed out that the team can deal with increasing information complexity more efficiently, but overload will occur when a certain point of complexity exceeds its information processing capacity. Wang et al. (2014) showed that a highly complex website resulted in less task completion, more eye fixations, and longer eye fixations compared to a less complex website. When people perform complex tasks on a highly complex website, they suffer from a high perceptual load and do not have enough capacity to process irrelevant stimuli. Therefore, the information complexity will have a positive effect on information overload.

Information overload will have an impact on the behavior intention. Furner et al. (2016) found an inverted U-shaped curve between information load and purchasing intentions, when the information load continues to increase to a reasonable and moderate point, it will promote the user’s purchase intention, while the purchase intention will reduce after the information load exceeding the moderate point. Studies of healthy behavior showed that information overload is negatively related to the performance of health behaviors and behavioral intentions. For example, those who perceived higher information overload are less likely to perform regular medical checkups and cancer screening, such as colonoscopy and mammography (Han et al., 2007; Jensen et al., 2014), and have less intention to engage in cancer prevention behavior (Niederdeppe et al., 2014; Jensen et al., 2020).

The attitude of physicians is also an important determinant of antibiotic prescription behavior (Teixeira Rodrigues et al., 2013), Hassali et al. (2015) applied the KAP theory and found that most physicians have a certain understanding of the use of antibiotics for upper respiratory tract infections and have a positive attitude toward the use of antibiotics while they are worried that the actual prescription behavior will be affected by the patients; Walker et al. (2001) found that physicians’ attitude and behavior control are important determinants of the intention to prescribe antibiotics based on TPB. They believed that intervention design should start with prescription motivation, and pointed out that it is needed to develop TPB or other psychological models to promote the improvement of antibiotic prescription behaviors; Liu et al. (2019) found that physicians with a recognized attitude towards antibiotics are often more willing to prescribe antibiotics and less willing to reduce antibiotic prescriptions.

Information overload can also affect information behavior intention through attitude. Swar et al. (2017) found that online health information overload affects users through attitudes, perceived information overload has a positive impact on the psychological illness of information seekers, and reduces the behavior intention of continuing to search for health information. Crook et al. (2016) found that perceived information overload of health knowledge will negatively affect the individual’s attitude towards health information, and the behavior intention to share health information will also decrease.

When the information complexity of the AST reports exceeds a certain level, physicians will perceive information overload, and the attitude towards the information provided by the AST reports may change. Eventually, the behavior intention to prescribe antibiotics based on the AST reports will be affected.

Based on the discussion above, it is hypothesized that the information complexity of AST reports can influence the physician’s intention to prescribe antibiotics based on the AST report through the mediating effect of information overload and attitude.

## Materials and Methods

### Study Design and Participants

A cross-sectional study was conducted in a teaching tertiary hospital in Ganzhou, Jiangxi, China in July 2021.

Strains in the research hospital were identified using the VITEK2-compact microbial identification system (BioMérieux, France). *In vitro* susceptibility testing was carried out by Kirby-Bauer disk diffusion, the interpretation standards, and quality control requirements were followed the CLSI guidelines (CLSI 2021).

The physician inclusion criteria were as follows: 1) Physicians who have the right to prescribe antibiotics or senior interns with extensive prescribing-assistant experience in prescription; 2) All physicians on duty during the on-site investigation; 3) Physicians who can understand and fill out questionnaires.

Our investigation was combined with the task of monitoring the rational use of drugs in the research hospital, questionnaires were issued to all physicians on duty during the investigation. A total of 411 questionnaires were collected, including 306 physicians with the prescription rights (76.12%) and 96 senior interns (23.88%), nine missed prescription information. The participants’ ages ranged from 20 to 58 years old (33.19 ± 7.94), 31 missed age information; a majority of the sample was males (270, 67.50%), 11 missed gender information.

### Measurements

All measurements used in this study were based on established existing scales. English original scales were translated and checked for back-translation by both Chinese- and English-speaking researchers. The participants were asked to respond on a 5-point scale ranging from 1 “strongly disagree” to 5“strongly agree.”

#### Information Complexity

Information complexity of the AST report was measured by three items adapted from Huang (2000) and Li (2016) with good reliability in this study (α = 0.825). The items are: “The amount of information in the AST report is large,” “The information in the AST report has many aspects” and “The information in the AST report is complex.”

#### Information Overload

Information overload on the AST report was measured by four items adapted from Chen et al. (2009) and Swar et al. (2017) with good reliability in this study (α = 0.846). The items are: “I am burdened in handling too much information in the AST report,” “I found little information in the AST report relevant to my prescription,” “I am not sure to choose antibiotics due to many antibiotics in the AST report” and “I could handle all the information effectively in the AST report.”

#### Attitude

Physicians’ attitude towards the information in the AST report was assessed using a five-item measure adapted from Stephens and Rains. (2011) with good reliability in this study (α = 0.904). Participants rated the degree to which they felt that AST report information was helpful to them to prescribe antibiotics, b) a valuable resource, c) important for prescribing, d) a good idea for clinicians, e) a waste of time (reverse coded).

#### Behavior Intention

Three items created for this study were used to measure physicians’ behavior intention to prescribe antibiotics based on the AST report with good reliability in the current study (α = 0.708). The items are: “I intend to prescribe antibiotics based on the AST report,” “I intend to prescribe antibiotics based on experience first, and then adjust the prescription based on the AST report” and “I intend to prescribe antibiotics listed in the AST report.”

### Statistics Analysis

In this study, IBM SPSS Statistics version 24 and Amos 24.0 was used to complete all the data analysis and processing. Firstly, as all the data were collected through a self-reported questionnaire, a test of common method bias was first conducted with AMOS. Secondly, descriptive analysis was used to describe the general characteristics of the study population. Thirdly, Pearson correlation analysis was used to examine the correlations between information complexity, information overload, attitude, and behavior intention. Lastly, the multiple mediating effects was tested using the SPSS PROCESS macro developed by Hayes (2013). Model 6 is a serial multiple mediator model. A *p*-value of 0.05 was considered statistically significant. We set the bootstrap confidence interval (CI) at 95%, and the number of bootstrap samples was 5,000. If zero was not included in the interval of 95% CI, it indicated that the mediating effect was significant.

## Results

### Test of Common Method Bias

To test for any potential common method bias, a test procedure suggested by the researcher was adopted a confirmatory factor analysis with a single factor explaining all the variance in the research data revealed a poor model fit (χ2/df = 13.462, RMSEA = 0.174, TLI = 0.553, CFI = 0.617), indicating that there were no significant biasing effects on the subsequent data analysis.

### Descriptive Statistics and Correlation Analysis

The means, standard deviations, and bivariate correlations among study variables are presented in Table 1. Correlation analysis showed that information complexity was significantly positive correlated with information overload (*r* = 0.20, *p* < 0.01), attitude (*r* = 0.41, *p* < 0.01), and behavior intention (*r* = 0.41, *p* < 0.01); Information overload was significantly negatively correlated with attitude (*r* = −0.12, *p* < 0.05) and significantly positive correlated with behavior intention (*r* = 0.15, *p* < 0.01); Attitude was significantly positive correlated with behavior intention (*r* = 0.42, *p* < 0.01).

**TABLE 1:** Descriptive statistics and correlation analysis of information overload and attitude between information complexity and behavior intention.

Variable	Minimum	Maximum	Mean	Std. Deviation	1	2	3	4
1.Information complexity	2.00	5.00	4.10	0.60	1			
2.Information overload	2.00	5.00	3.30	0.65	0.20**	1		
3.Attitude	2.40	5.00	3.98	0.43	0.41**	−0.12*	1	
4.Behavior intention	2.60	5.00	4.04	0.49	0.39**	0.15**	0.42**	1

**p < 0.01: *p < 0.05.

### Mediating Model Analysis

Regression analysis (Table2) showed that information complexity of the AST report was positively associated with the information overload (β = 0.222, *p* < 0.001)and attitude (β = 0.324, *p* < 0.001); information overload was negatively associated with attitude (β = 0.-0.141, *p* < 0.001); at the same time, information complexity, information overload and attitude were all positively associated with the intention to prescribe antibiotics based on the AST report (β = 0.173, *p* < 0.001; β = 0.114, *p* < 0.001; β = 0.125, *p* < 0.01).

**TABLE 2:** Multiple regression analysis of information overload and attitude between information complexity and behavior intention.

Regression model		Model fit			Coefficients	
Dependent variable	Independent variable	R	*R* ^2^	F	β	t
Behavior Intention	Information complexity				0.173	4.326***
	Information Overload	0.503	0.253	45.914	0.114	3.375***
	Attitude				0.402	7.315***
Attitude	Information complexity	0.459	0.210	54.322	0.324	10.045***
	Information Overload				−0.141	−4.769***
Information Overload	Information complexity	0.204	0.041	17.675	0.222	4.204***

*** *p*<0.001.

**FIGURE 1:**
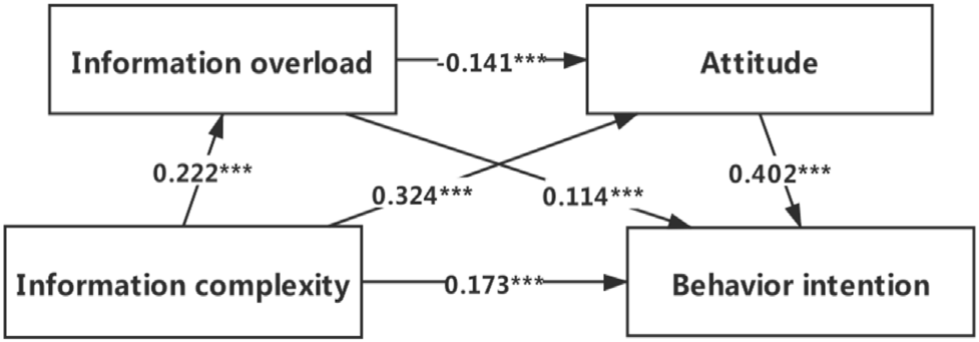
Serial multiple mediating model of information complexity, information overload, attitude and behavior intention. *** p < 0.001.

The serial mediation analysis (Table 3) showed that the total effect of information complexity on behavior intention was 0.315 (*p* < 0.001) and information complexity had a significant direct effect on behavior intention (effect = 0.173; SE = 0.044; Boot 95%CI: LL = 0.089, UL = 0.260). In addition, all three indirect paths were also significant. The first indirect pathway was that information overload significantly mediated the effect of information complexity on behavior intention (effect = 0.025; SE = 0.011; Boot 95%CI: LL = 0.008, UL = 0.050). The second indirect pathway was that the effect of information complexity on behavior intention was significantly mediated by attitude (effect = 0.130; SE = 0.025; Boot 95%CI: LL = 0.086, UL = 0.180). The third indirect pathway showed that information overload and attitude played a masking effect between information complexity and behavior intention (effect = −0.013; SE = 0.004; Boot 95%CI: LL = −0.023, UL = −0.005). The mediating model and the values of the pathways were presented in Figure 1.

**TABLE 3:** Multiple Mediating Effects Test of information overload and attitude between information complexity and behavior intention.

	Effect	BootSE	BootLLCI	BootULCI	Relative effect
Total effect	0.315	0.045	0.225	0.403	−
Direct effect	0.173	0.044	0.089	0.260	−
Total indirect effect	0.143	0.027	0.094	0.195	−
Indirect 1	0.025	0.011	0.008	0.050	14.45%
Indirect 2	0.130	0.025	0.086	0.180	75.14%
Indirect 3	−0.013	0.004	−0.023	−0.005	7.51%

AbbreviationIndirect 1, information complexity → information overload → behavior intention; Indirect 2, information complexity → attitude → behavior intention; Indirect 3, information complexity → information overload → attitude → behavior intention. Effect, standardized regression coefficient; BootSE, bootstrapping standard error; BootLLCI, bootstrapping lower limit confidence interval; BootULCI, bootstrapping upper limit confidence interval.

Note: Relative effect = |Indirect Effect/Direct Effect|*100%.

## Discussion

### Main Findings

This study investigated the influence of AST report information complexity on the intention to prescribe antibiotics based on AST report, as well as the underlying mechanism of information overload and attitude mediating this association. We found that the information complexity of the AST report will have a direct positive impact on physicians’ behaviour intention to prescribe antibiotics based on the AST report, information overload and attitude will have impacts among them. Our hypotheses have been confirmed, which provides new evidence for the impact of information complexity on behaviour intentions and enriches the influencing factors of antibiotic prescription behaviour intention.

### The Direct Effect of Information Complexity on Behavior Intention

The results showed that the information complexity of the AST report has a positive effect on the behavior intention. It is similar to the results of Donovan and Rossiter (1982) and Huang (2000) that the information complexity will have a beneficial effect on the desire to stay longer in conventional retail environments because consumers expected greater variety of goods available, thereby the purchase intention increased, although this may be an impulse purchase. The positive effects of our research might be due to the self-efficacy of most participants who have high level of education and clinical experience. The previous study has shown that self-efficacy may affect a person’s information-seeking behavior, and people with higher self-efficacy will seek more information (Josephine et al., 2017). Therefore, when the AST report provides more information, physicians with higher self-efficacy will continue to seek more information provided by the report as a reference to prescribing antibiotics. However, most previous studies believe that information complexity has a negative impact on behavior intention. Both Huffman and Kahn (1998) and Li (2016) found that the increased complexity of online review information will make consumers confused and reduce their purchase intention. Our research provides new evidence for positive effects. It shows that physicians intend to seek more information for prescribing antibiotics, the possible reason is that this can increase their confidence in making antibiotic prescription decisions by more evidence from AST reports. Karr-Wisniewski and Ying (2010) believed that humans tend to make satisficing decisions versus optimal decisions due to their search and information processing capacity. Although processing more information will decrease decision-making performance, the confidence and satisfaction of decision-makers will increase (Charles and O’Reilly, 1980). The extrinsic factors can also explain the positive effect between information complexity and behavior intention. Chinese physicians experience heavy expectations from patients and the pressure of the healthcare system (Warreman et al., 2019), which may induce physicians to be more cautious in prescribing antibiotics especially in our research institute, a large teaching tertiary hospital. They may tend to seek more information to make their prescriptions more evidence-based to meet patient expectations or avoid patient complaints and potential conflicts.

### The Mediation Effect of Information Overload and Attitude

The results showed that information overload and attitude mediated the association between information complexity and the intention to prescribe antibiotics based on the AST report, and this mediation includes three pathways: the independent mediating effects of information overload and attitude and the chain masking effect of information overload and attitude. This also verifies that information overload and attitude are the influencing factors of behavioral intention.

Since the AST report includes various clinical and laboratory information, physicians need to process the information in the AST report and make the best choice from many antibiotics based on the patient’s situation, and sometimes need to consider the consistency of antibiotics reported and hospital inventory, physicians are likely to perceived overload of these complex information in their busy work, information complexity will positively affect information overload has been verified in previous studies (Otondo et al., 2008; Li, 2016). As mentioned above, when information overload does not exceed a reasonable moderate point, its increase will promote behavior intention (Furner et al., 2016). Therefore, a certain degree of information overload can promote the association between information complexity and behavior intention. Some studies found that information complexity can cause doubt, confusion, and distrust in the process of information seeking (Sarah et al., 1995; Park, 2019), but we found that the increase in information complexity will positively affect physicians’ attitudes towards the information in the AST report. This may be because when the amount of information is larger and more complex, the number of clues that users can use for decision-making is greater (Chebat et al., 2003). There are many uncertainties in clinical decision-making, so current valid evidence is needed to support clinical decisions (Hall and Walton, 2004). The AST report is important evidence for antibiotic prescription decisions. Physicians rely on the AST report to identify pathogens and make targeted antibiotic prescriptions. The more information provided in the AST report, the greater the number of clues that physicians believe can be used for reference. Also, the more complex the AST results may represent the more complicated or more serious infection symptoms, physicians will be more cautious, and will pay more attention to the information in the AST report, and are willing to find useful information from the AST report to prescribe rational antibiotics, which means that the willingness to prescribe antibacterial drugs based on the AST report will increase. If the physician has a high positive attitude to the AST report, he will think that the AST report will be more helpful to prescribe antibiotics accurately and reasonably when the more information in the AST report. Therefore, the attitude also can promote the association between information complexity and behavior intention.

We also found that information overload and attitude play a masking effect between information complexity and behavior intention, the influence of information complexity on behavior intention changes from positive to negative. This may be because there is a negative correlation between information overload and attitudes. The increase in information overload caused by the information complexity may make physicians feel confused and suspicious about the information in the AST report (Niu et al., 2020). It will lead to a decrease in the willingness of doctors to obtain information from the AST report, which means that the intention to prescribe antibiotics based on the AST report will decrease. This can be explained by the “inverted U-shaped curve” (Jackson and Farzaneh, 2012) between the amount of information and decision-making behavior. The doctor’s intention to prescribe antibiotics based on the AST report will increase as the amount of information in the AST report increases, but when the complexity of the information exceeds a certain threshold, it will have a hindering effect on behavior intention. The time pressure of physicians at work can also explain this negative impact, especially under the high degree of overload in a large tertiary hospital, more information will become obstructive rather than useful when physicians need to process complex information in a limited time (Hall and Walton, 2004), their attitudes towards information will become negative as a result. Granato (1993) have found that physicians are more inclined to prescribe antibiotics empirically because the AST report provides more information than their needs. Therefore, it is suggested that there is an optimal range of information in which the physicians will adequately process and evaluate the information (Sicilia and Ruiz, 2010), without causing negative attitudes and influence on behavior intention from the pressure of information overload caused by excessive information.

Our research has some limitations. First, the data is collected through self-reporting. This is a cross-sectional survey. In the future, longitudinal or experimental designs will be used to reveal the causality of physicians’ behavior intentions for prescribing antibiotics based on AST reports. Second, we surveyed in only one general hospital, which limits the scope of promotion. In the future, data will be collected in multiple centers to expand the sample size and make the results more representative. Finally, this study only considers the complexity as one of the information characteristics, more information factors and possible individual factors will be considered in future studies.

## Conclusion

The information complexity of the AST report can have a direct impact on the behaviour intention of physicians prescribing antibiotics based on the AST report, information overload and attitude can also promote this effect. It shows that in a complex clinical environment, especially for the complex behaviour of prescribing antibiotics, if the information complexity of the AST report is appropriately increased, more information can be provided as a reference for decision-making. Then physicians will perceive a certain degree of information overload which can help improve doctors’ attitudes towards AST report information, thereby promoting doctors to prescribe antibiotics based on the AST report. It is enlightened that we should provide physicians with sufficient information as a prescription reference without increasing the burden on them. At the same time, it is also needed to conduct publicity and standardized training for physicians, and strengthen communication between laboratories and physicians to help doctors interpret AST reports better and faster. So that the AST report can play the important role in the prescription of antibiotics.

## Data Availability

The raw data supporting the conclusion of this article will be made available by the authors, without undue reservation.

## Abbreviations

AST, antimicrobial susceptibility testing; AMR, Antimicrobial resistance; Effect, standardized 383 regression coefficient; SE, standard error; BootLLCI, bootstrapping lower limit confidence interval; 384 BootULCI, bootstrapping upper limit confidence interval; LOS, length of stay; OR, odds ratio; 95% 385 CI, 95% confidence interval.
